# The Impact of Epigenetic Modifications in Myeloid Malignancies

**DOI:** 10.3390/ijms22095013

**Published:** 2021-05-09

**Authors:** Deirdra Venney, Adone Mohd-Sarip, Ken I Mills

**Affiliations:** Patrick G Johnston Center for Cancer Research, Queens University Belfast, 97 Lisburn Road, Belfast BT9 7AE, UK; dvenney01@qub.ac.uk (D.V.); a.mohdsarip@qub.ac.uk (A.M.-S.)

**Keywords:** Epigenetics, MDS, MPN, AML, DNMT3A, TET2, ASXL1, IDH1, IDH2, EZH2

## Abstract

Myeloid malignancy is a broad term encapsulating myeloproliferative neoplasms (MPN), myelodysplastic syndrome (MDS) and acute myeloid leukaemia (AML). Initial studies into genomic profiles of these diseases have shown 2000 somatic mutations prevalent across the spectrum of myeloid blood disorders. Epigenetic mutations are emerging as critical components of disease progression, with mutations in genes controlling chromatin regulation and methylation/acetylation status. Genes such as DNA methyltransferase 3A (*DNMT3A*), ten eleven translocation methylcytosine dioxygenase 2 (*TET2*), additional sex combs-like 1 (*ASXL1*), enhancer of zeste homolog 2 (*EZH2*) and isocitrate dehydrogenase 1/2 (*IDH1/2*) show functional impact in disease pathogenesis. In this review we discuss how current knowledge relating to disease progression, mutational profile and therapeutic potential is progressing and increasing understanding of myeloid malignancies.

## 1. Introduction

Haematopoiesis is a tightly regulated process involving gene expression control that guides conversion from progenitor cells to terminally differentiated haematopoietic cells. During leukaemogenesis, processes such as self-renewal, differentiation and cell expansion are disrupted, resulting in accumulation of immature blast cells. Initial cytogenetic studies in leukaemia identified chromosomal translocations which generate oncogenic fusion proteins mainly altering transcriptional regulators [1]. Subsequently, recurrent somatic mutations were discovered in genes encoding regulators of cellular differentiation and signalling that were required for malignant transformation. These classical models in leukaemia research together with the original two-hit hypothesis have been unsuccessful in explaining the distinctive characteristics of decreased differentiation in leukaemia [2]. Myeloid malignancies encapsulate a group of disorders caused by clonal expansion of haematopoietic stem/progenitor cells including acute myeloid leukaemia (AML), myelodysplastic syndrome (MDS) and myeloproliferative neoplasms (MPN) (polycythaemia vera, essential thrombocythemia and primary myelofibrosis). MPN and MDS possess the ability to transform into secondary AML.

The interplay of epigenetic processes, DNA methylation and histone modifications, determine if a (set of) gene(s) is transcriptionally expressed or silenced. Examples include methylation of CpG islands, (de)methylation, and (de)acetylation of histones. These processes regulate chromatin conformation and in turn regulate gene expression. Studies have revealed the essential role of deregulated epigenetic processes in AML [3,4]. Because epigenetic modifications are inherently reversible and dynamic, deregulated epigenetic mechanisms might provide a path towards targeted therapies via inhibitors of proteins that modify histones or DNA methylation. Chromatin structure has a large impact on the regulation of gene expression, and a lot more can be discovered about how individual epigenetic marks are set up and then maintained throughout DNA replication and cell division. Chemical modification of DNA or chromatin-associated proteins and histones have a major effect on chromatin structure and gene expression.

In this review, we take advantage of evidence from a range of somatic mutations frequently occurring in chromatin and epigenetic modifiers as well as explore the relationship between DNA methylation and histone modification. We will describe the relationship between these modifiers and leukaemogenic pathways leading to decreased cell differentiation and deviant self-renewal and proliferation. We then discuss how these data based on epigenetic modifiers have, and will continue to, lead to innovative epigenetic therapeutics. Finally, we address how understanding the combinatorial factors leading to AML will enable us to decipher epigenetic reprogramming and understand the mechanisms of gene regulation.

## 2. Current Knowledge of Genetic Profiles for Myeloid Malignancies

Mutations impacting myeloid malignancies can be divided by five classes: spliceosome components (e.g., Splicing factor 3B subunit 1 (*SF3B1*)), epigenetic regulators (e.g., DNA methyltransferase 3A (*DNMT3A*),), signalling pathway proteins (fms-like tyrosine kinase 3 (*FLT3*), Janus kinase 2 (*JAK2*)), transcription factors (CCAAT enhancer-binding protein alpha (*CEBPA*), runt-related transcription factor 1 (*RUNX1*)) and tumour suppressor proteins (Tumour protein p53 (*TP53*)) [5]. The last four decades have showed varying rates of development for clinical treatment for these myeloid disorders, encompassing chemotherapy and tyrosine kinase inhibitors (TKI’s) to more modern therapies which include epigenetically active inhibitors like histone deacetylase inhibitors (HDACi) and DNA methyltransferases (DNMT) that have an impact on chromatin structure and accessibility [6,7].

Multiple somatic mutations, which occur in genes encoding regulators of epigenetic modifiers, have shown to alter epigenetic and chromatin remodelling activity of the cell. Yet, the original two-hit hypothesis for myeloid progression does not cover all disease alleles. Mutant alleles that do not conform to either class have shown to promote epigenetic modifications and include alterations in *DNMT3A* and ten eleven translocation methylcytosine dioxygenase 2 (*TET2*). These data suggest there is an ever increasing need to explore the expanding biological and clinical relevance of epigenetic alterations in myeloid malignancies.

**Figure 1:**
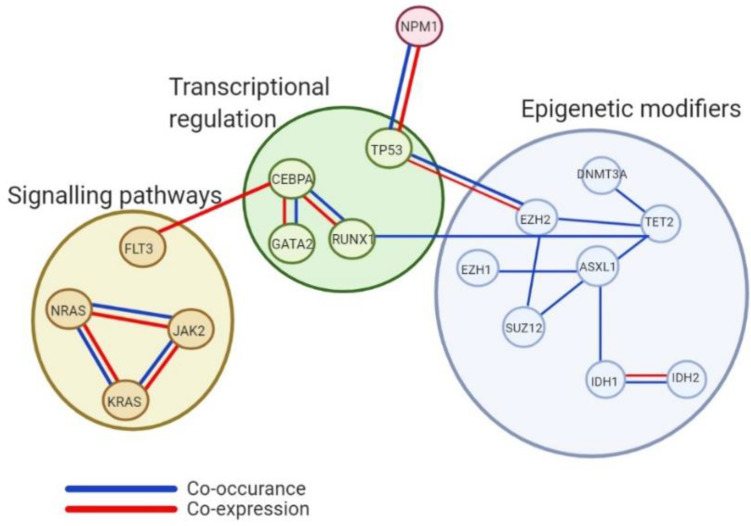
Analysis of co-occurrence and co-expression of epigenetic genes and other cellular regulatory processes. Interactions shown between genes considered to possess epigenetic modification processes and other cellular processes like transcriptional regulation and signalling and kinase pathways highlighting the intricate pathway association needing accounted for in the progression of knowledge around mutational profiles within myeloid malignancies.

These epigenetic alterations influence a range of cellular processes having further impact outside of the epigenome through control of chromatin and gene expression. Indicating that chromatin and epigenetic modifiers embody a cluster of mutations essential for leukaemic development. It is widely accepted that epigenetic alterations rarely occur alone but culminate from a large profile of concomitant mutations (Figure 1).

Recurrent cytogenetic events and somatic variants revealed from sequencing analyses uncovered 9 genetic classes off AML [5]. Deep sequencing studies across 200 patients by The Cancer Genome Atlas (TCGA) uncovered a heterogeneous disease with approximately 2,000 somatically mutated genes [8]. Majority of these recurrent cytogenetic events and somatic mutations play an important role in prognosis [5,9]. Some the most frequent somatic mutations have been detected in both MDS and MPN, as well as healthy individuals with age-related clonal haematopoiesis, thus associated with substantial risk for progression of myeloid malignancies [10,11]. The AML karyotype has been well documented in the European LeukemiaNet (ELN) risk stratification guide was revised in 2017 to outline the favourable-risk (FR), intermediate-risk (IR) and adverse-risk (AR) profiles. ‘Favourable-risk’ cytogenetic abnormalities include Acute Myeloid Leukaemia- Eight Twenty-Two (*AML-ETO*), also known as RUNX1 Translocation Partner 1 (*RUNX1T1*), Pro Myelocytic Leukaemia- Retinoic Acid Receptor alpha (*PML-RARA*) and Nucleophosmin 1 (*NPM1*) mutations [12,13]. Whereas most deletions (*−5/del(5q*)), inv(3)/t (3;3), fms-like tyrosine kinase 3- internal tandem duplications (*FLT3-ITD*) mutations and complex karyotypes are deemed as having an adverse prognostic outcome [1,14]. The abnormalities in intermediate-risk groups include cytogenetic abnormalities not listed in “favourable-risk” or adverse-risk groups or those with double mutations in *CEBPA* or no mutations in *NPM/FLT3-ITD*.

Functional investigations have highlighted two main classes of leukaemic disease alleles that contribute to the haematopoietic transformation targeting either signal transduction or epigenetic control in myeloid malignancies. Class I alleles, such as *FLT3* and *JAK2*, have been shown to confer advantage to growth due to irregular activation and proliferation of cell signalling pathways (*STAT*, *PI3K* and *RAS-MAPK*) [15]. While class II alleles like mixed-lineage leukaemia (*MLL*) and *CEBPα* alter transcriptional targets used in haematopoietic differentiation and maturation [9,16].

## 3. Alterations in DNA Methylation

Methylation of DNA encompasses the addition of a methyl group to the 5-carbon position of cytosines in CpG dinucleotides producing 5-methylcytosine [17]. This is established by DNA methyltransferases (DNMTs). Altered DNA methylation patterns have been reported in the regions of CpG islands (gene promoters). The association of hypermethylation and hypomethylation of cytosines in CpG islands are pertinent in gene regulation. CpG islands are DNA methylation regions contained within promoters, they regulate expression of genes via transcriptional silencing of the corresponding gene [18].

**Figure 2:**
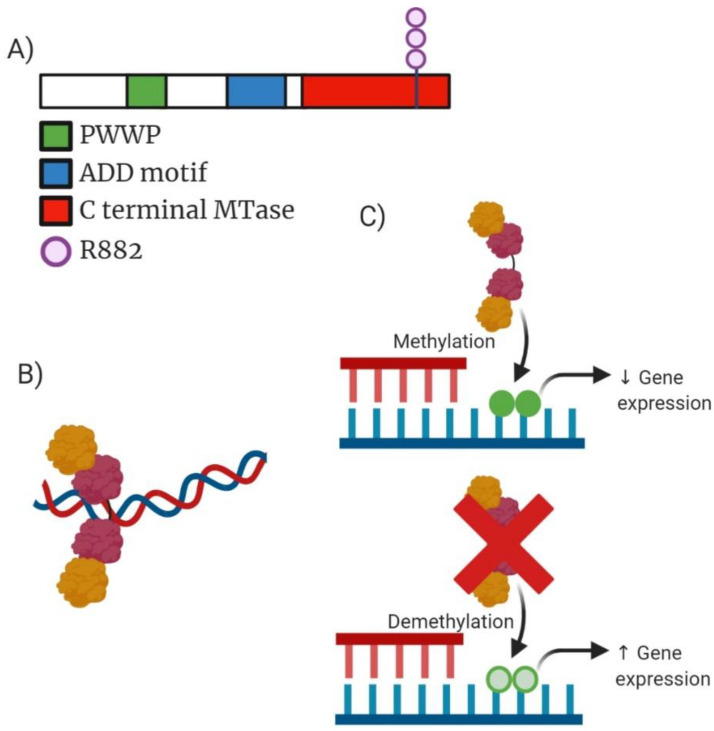
Analysis of *DNMT3A* molecular structure, active tetramer structure, mechanism of action and drug target action. (**A**) Domain structure of mammalian *DNMT3A* enzyme consisting of 912 amino acid residues. Green highlighting PWWP domain which is required for directing DNA methylation. Blue highlighting ADD (ATRX-DNMT3A-DNMT3L) motif responsible for mediating protein–protein interaction to transcription factors. Red highlights the C-terminal MTase domain contains highly conserved regions of C5-DNA methyltransferases. Additionally, highlighted is the structural position of the most common mutation occurring within *DNMT3A* (R882). (**B**) Active tetramer formed from two *DNMT3A* and two *DNMT3L* molecules resulting in an increased affinity for DNA causing more efficient methylation. (**C**) Mechanism of action for both wild-type (WT) *DNMT3A* and the truncated mutant *DNMT3A*. WT *DNMT3A* causing methylation which can reduce expression of gene expression compared to mutant *DNMT3A* which can often result in increased expression of genes depending on the context of the cellular function occurring.

Mutations occurring in *DNMT3A* is one of the most common mutations displayed in AML with ~20% of de novo patients displaying this aberration [19]. The *DNMT3A* gene encodes a methyltransferase that adds a methyl to the cytosine residue of CpG dinucleotides creating a C5 methylcytosine (5mC) (Figure 2A). Mutations in the *DNMT3A* gene were first identified and described by Ley et al. in 2010 after sequencing an AML patient who had a normal cytogenetic profile [19]. Increased methylation status was observed in *DNMT3A* mutants leading to reduced expression of downstream genes [20]. *DNMT3A* and its co-factor, *DNMT3L*, form an active tetramer with an increased affinity for DNA resulting in more efficient methylation (Figure 2B) [21]. Majority of mutations in the *DNMT3A* gene resulted in either nonsense/frameshift alterations which caused premature truncation of the protein product or an amino acid substitution at Arginine 882 (R882) (Figure 2A). In total, 65% of mutations in this amino acid are heterozygous missense mutations that exert a dominant effect over wild-type (WT) *DNMT3A* genes. R882 mutations have previously been shown to compromise methyltransferase activity resulting in increased self-renewal activity of haematopoietic stem cells (HSC) (Figure 2C) [22]. Koya et al. has shown that R882 mutants can dimerize with WT *DNMT3A* molecules but fail to form active tetramers resulting in a significant decrease in methyltransferase activity [23]. *DNMT3* mutant R882 can interact with Polycomb Repressive complex 1 (PRC1) resulting in downregulation of haematopoietic differentiation genes like *CEBPα*, stunting cells in an immature state which still retain the capacity for self-renewal causing long-term expansion of HSC [24]. Crucial findings in highlighting the role of *DNMT3A^R882H^* mutations in AML chemoresistance showed the importance of aberrant recruitment of histone chaperone SPT-16 resulting in attenuated nucleosome eviction and chromatin remodelling as a result of exposure to anthracycline [25].

Many epigenetic alterations rarely occur alone in myeloid malignancies. Mutant *DNMT3A* is frequently associated with mutations in *NPM1*, *FLT3* and isocitrate dehydrogenase 1/2 (*IDH1/2*), working together to cooperatively corrupt leukaemic progenitor cells. Patients who present with this collection of mutations tend to display a higher percentage of bone marrow blast cells while also experiencing a shorter time of event free survival (EFS) [26]. Patients with higher white blood counts (WBC) and platelets have been reported versus patients without the aberration. *DNMT3A* mutations display promiscuity in several myeloid malignancies and influence disease progression in MPS, MDS and de novo AML. Within AML patients it has been shown that *DNMT3A* mutations are enriched in those classified as M4/M5 AML by classification guidelines outlined by the French American-British classification system [27].

DNA methylation profiling provides information regarding diagnostic classification, prognostic stratification and therapeutic guidance in myeloid malignancies. Alterations in DNA methylation have been widely reported in most cancers, the result of these alterations promotes oncogenesis due to changes in gene expression [28]. Modern advancements in methylome analysis, e.g., microarray assays, have reduced time, cost and reproducibility of DNA methylation analysis, resulting in it becoming a reliable method in clinical diagnosis of myeloid malignancies [29,30,31]. It has been previously highlighted that known global methylation patterns assist in genetic characterisation of AML subgroups; Figueroa et al. reported distinct DNA methylation profiles which can be linked to characterisation of AML profiles indicating a clear link between methylation profiling and disease diagnosis leading to clinical decision making on treatment options [32,33].

## 4. Alterations in DNA Hydroxymethylation

**Figure 3:**
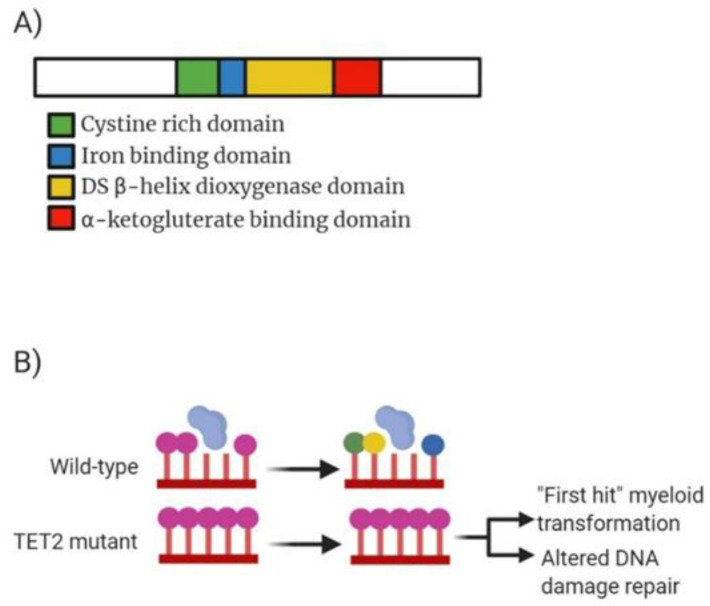
Analysis of *TET2* molecular structure, mechanism of action and drug target action. (**A**) Domain structure of mammalian *TET2* enzyme consisting of 2002 amino acid residues. Green highlighting a cystine rich domain comprised of two sub-domains which modulate chromatin targeting by *TET* proteins. Blue highlighting the iron binding domain which interacts with double stranded β-helix domain (yellow) and α-ketoglutarate binding domain (red) to form a core catalytic region. DSBD also contains a low complexity insert whose function remains unclear. (**B**) Mechanism of action for both wild-type *TET2* and the mutant *TET2*. WT *TET2* causing conversion of 5-mC (purple circle) into 5-hmC (green), 5-fC (yellow) or 5-caC (blue). Mutated *TET2* lowers expression resulting in overexpression of 5-hmC causing control over genes being switched on or off.

The oxidation process of 5-methylcytosine to produce 5-hydroxymethylcytosine (5hmC) is catalysed by the TET-enzymes which is dependent on alpha-ketoglutarate (α-KG). *TET2* was first discovered in 2009 with inactivating mutations occurring in MPM and AML patients, Delhommeau et al. accomplished this through mapping structural DNA rearrangements in chromosome 4q24 [34]. *TET2* is a member of the *TET* family of proteins with mutations of this gene occurring in 15% of myeloid malignancies (Figure 3A). *TET2*-associated mutations have been observed in AML patients and are linked with reduced levels of 5hmC [35] as well as contribute to a poor prognosis in intermediate-risk AML [36,37].

Genetic studies into *TET2* have revealed the link between alterations in DNA methylation and tumour metabolism. Mutations in the widely recognised tumour suppressive *TET2* gene have been shown to be sufficient to instigate myeloid malignancies in mice [38]. Mutations to the *TET2* gene have been shown to include frameshift, early stop codons, deletions and AA substitutions highlighting a widely diverse profile for potential alterations. Majority of patients carry heterozygous *TET2* mutations however a small proportion of patients present with a total homozygous loss of *TET2*. Previous work sequencing the coding exons of the gene by Jankowska et al. highlighted the association of homozygous mutations with uniparental disomy 4q compared to heterozygous mutations associated with lack of chromosomal lesions [39]. Studies have been reported on the mutational role of *TET2* on progression of disease and suggested an involvement in early clonal mutations due to the presence of alterations in both myeloid and lymphoid progenitor compartments. Papaemmanuil et al. strengthened this by discovering a high allele frequency in *TET2* mutations indicating that they occur as part of the “first hit” of mutational development in leukaemogenesis [5]. Once mutated, *TET2* produces decreased levels of 5-hmC resulting in the unblocking of methyl-binding proteins (Figure 3B). Normal deposition of 5-hmC in CpG dinucleotides results in increased gene expression for those regions.

*TET2* mutations have been observed to co-occur with *NPM1*, *FLT3-ITD*, *JAK2*, *ASXL1*, calreticulin (*CALR*), *SF3B1* and *RUNX1* revealing the range of epigenetic connections to cellular processes amongst various myeloid malignancies [40]. It is worth noting that mutations in *TET2* are mutually exclusive together with Wilms’ tumour suppressor gene 1 (*WT1*), in 30–50% of AML patients. These patients present with a distinct AML subtype displaying dysregulated DNA hydroxymethylation. AML patients with *TET2* mutations have a high WBC along with low platelet counts. Patients aged over 65 years old also experience low EFS (8.9 months vs. not reached) suggesting a role for *TET2* as a prognostic biomarker [41]. AML patients defined as favourable risk (containing *CEBPA* and/or *MPN1* without *FLT3-ITD*) by the European Leukaemia Net (ELN) will display worse EFS in the presence of *TET2* mutations (10 months vs. 41.3 months) [41]. Yildirim et al. defined associations between 5-hmC and gene regulation through the interaction with methyl-CpG binding domain protein 3 (*MBD3*) a member of the NuRD complex (Nucleosome Remodelling and Deacetylase complex). They found that genes marked with 5-hmC were recognised by *MBD3* and subsequently repressed [42]. The localisation of 5-hmC most often occurs in transcription sites and gene exons. The potential global impact of *TET2* loss on the genome can lead to the prediction that it would lead to increased methylation and reduction of 5-hmC levels.

**Figure 4:**
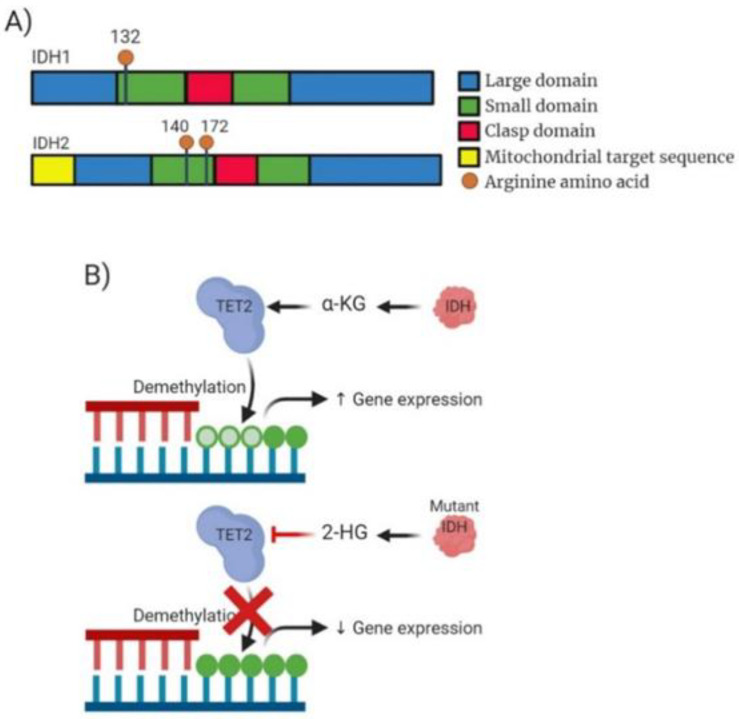
Analysis of *IDH* molecular structure, mechanism of action and drug target action. (**A**) Domain structure of mammalian *IDH1* enzyme consisting of 414 amino acid residues and *IDH2* consisting of 452 amino acids. Blue highlighting a large domain. Green highlighting the small domain. Red highlighting clasp domain which links other subunits together. *IDH2* also contains a mitochondrial targeting sequence. Additionally, highlighted is the structural position of the most common mutation occurring within *IDH1* (R132) and *IDH2* (R140/172). (**B**) Mechanism of action for both wild-type *IDH* and the mutant *IDH*. WT *IDH* causing production of α-KG enabling *TET2* to function in demethylation of DNA causing potential increased gene expression. Mutated *IDH* inhibits *TET2* function due to production of 2-HG expression often resulting decreased gene expression.

Isocitrate dehydrogenase 1 and 2 (*IDH1* and *IDH2*) proteins catalyse the conversion of isocitrate to α-KG. Mutations in genes encoding these *IDH1/2* enzymes are frequently observed in AML patients with normal cytogenetics. *IDH1/2* mutations result in aberrant metabolite 2-hydroxyglutarate (2-HG) [43] where it can compete with α-KG leading to *TET2* inhibition. *IDH1* mutations were discovered in 2008 by a sequencing project by Parsons et al. on human glioblastoma samples and since have been identified in AML patients by Mardis et al. [44,45]. *IDH2* mutations were identified based on consequent genomic studies of *IDH1* in AML [46]. Mutations in *IDH* proteins are found in 15–20% of AML cases [47]. Mutations in these genes are less common in MDS patients occurring in 3–5% of cases and 1–4% of MPN cases [48]. Mutational hotspots in *IDH1* have shown to occur at Arg132 while *IDH2* mutational hotspots occur at Arg140 more frequently than Arg172 [49] (Figure 4A). Ok et al. have shown that the persistence of *IDH* mutations lead to increased risk of relapse for patient’s post-treatment [50].

Patients presenting with *IDH* mutations are associated with distinct subsets of AML and normal/intermediate risk cytogenetic groups (27.1% of patients) suggesting that mutant *IDH* contribution can be linked to cell fate determination at the beginning stages of progenitor differentiation [26]. Clinical presentation includes older patients who present with higher platelet levels. Healthy individuals can also possess *IDH* mutations suggesting age-related clonal haematopoiesis; also suggesting the presence of *IDH* mutations in the beginning stages of leukaemogenesis and the requirement for concurrent mutations to enable AML progression. *IDH* mutations have been observed co-occurring alongside *NPM1* mutations and have better prognosis [40]. *IDH* mutations have shown to be mutually exclusive with *TET2* mutations. *TET2*, as described above, utilizes α-KG to enable the conversion of 5-mC into either 5-hydroxymethylcytosine (5hmC), 5-formylcytosine (5-fC) or 5-carboxylcytosine (5-caC) which is vital for the demethylation of DNA to control gene expression (Figure 4B). *IDH* mutations result in the levels of available α-KG being lowered in the cell due to the conversion to 2-HG. This action of *IDH* mutations exacerbates the impact of *TET2* mutations resulting in greater levels of *TET2*-dependent demethylation of DNA. This indirect interaction between *IDH* and *TET2* mutants results in increased global expression of 5-mC resulting in impaired DNA damage repair mechanisms or the promotion of myeloid malignancies.

## 5. Alterations in Histone/Lysine Methylation

The process of methylation on histone lysine residues can lead to mono-, di-, or trimethylation and is carried out by lysine methyltransferases (KMTs). These enzymes modify the affinity of reader proteins to methylated histones resulting in a context-dependent activation (H3K4, H3H36, and H3K79) or repression state (H3K9, H3K27, and H4K20) and whether mono-, di- or tri-methylation on the same lysine residue results in different functional consequences [51]. Post translational modifications (PTMs) are controlled by readers, writers and erasers which work in tandem to create, initiate or terminate biological signals within the cell [52]. The delicate balance of these activities controls the cellular regulation and any alterations in this balance can initiate dysregulation leading to human disease.

The lysine (K)-specific methyltransferase 2A (*KMT2A*) gene, also known as the mixed-lineage leukemia (*MLL*) gene, is a histone-lysine N-methyltransferase and leaves a mark of transcriptional activation by targeting H3K4. *MLL* is involved in translocations of approximately 10% of AML while mutations are frequently partial tandem duplications and found in 6% of de novo AML [53]. The normal *MLL* gene is located at 11q23 containing multiple motifs including AT hook DNA binding domains alongside domains for transcriptional activation and repression zinc finger domains [54]. *MLL* also contains a highly conserved SET domain which regulates homeotic (*Hox*) promoters [55]. The *MLL* gene contains an N terminal Menin binding domain. *Menin* protein acts a linker between the *MLL* gene and lens epithelium-derived growth factor (*LEDGF*), a chromatin binding protein [56]. Within the *MLL* gene there are 4 domains which are most commonly lost in the formation of *MLL* fusion proteins, these include the PHD finger, bromo-activation domain, activation domain and SET domain [57]. The majority of *MLL* rearrangements include the N terminal domain of the *MLL* gene and a C terminal domain of a translocation partner gene. Translocation partner genes can modulate transcription activity leading to activation of target genes or other diverse functions enabled by cytoplasmic/membrane bound partners [58]. There are more than 50 alternate translocation partners for the *MLL* gene that have been identified, each with unique biological properties. *MLL* has also been shown to interact with *RUNX1* to regulate the epigenetic function of *cis-*regulatory haematopoietic genes. *MLL* translocations result in fusion proteins lacking the SET domain and replaced by members of the super elongation complex such as AF9 and AF10. This subset of translocated *MLL* partners account for ~90% of *MLL* recombinations [59]. A member subunit of this complex is *DOT1L*, a KMT that targets H3K79. AML samples with translocated *MLL* demonstrate deregulated H3K79 methylation, leading to the expression of target genes, in particular the homeobox genes [60].

**Figure 5:**
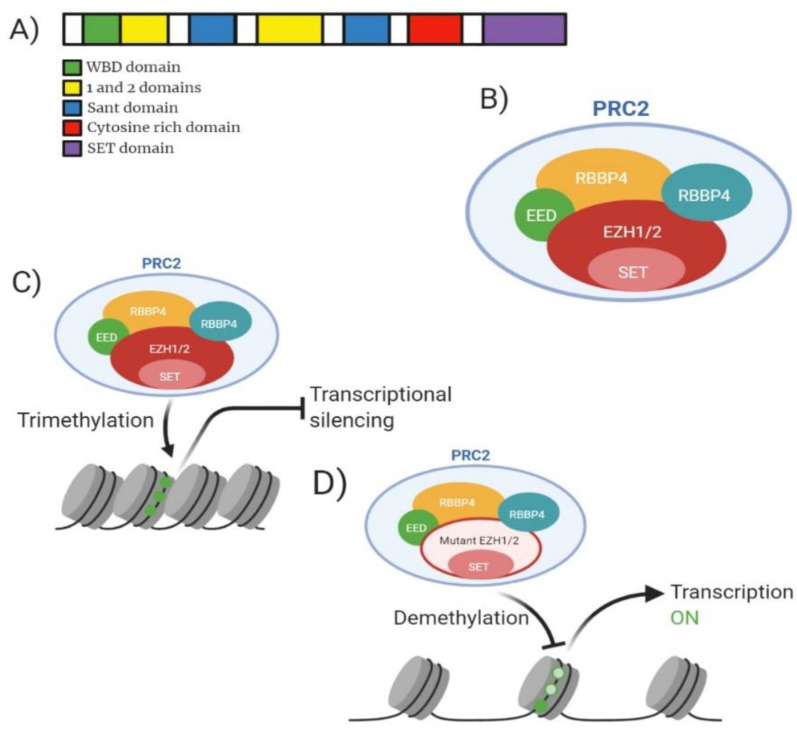
Analysis of *EZH2* molecular structure, PRC2 structure, mechanism of action for wild-type and mutant and drug target action. (**A**) Domain structure of mammalian *EZH2* enzyme consisting of 751 amino acid residues. Green highlighting WDB (WD-40 binding domain). Yellow highlighting domain ‘1’ which binds PHF1 and domain ‘2’ binding region for *SUZ12*. Blue highlighting SANT domain allowing interaction of chromatin remodelling proteins with histones. Red highlights cytosine rich domain. Purple highlighting catalytic SET domain. (**B**) Model of mammalian PRC2 complex with core subunits. (**C**) Mechanism of action for wild-type *EZH2* showing trimethylation of H3K27 resulting in gene transcription silencing. (**D**) Mechanism of action for PRC2 containing mutant *EZH2* showing reduced methylation of H3K27 resulting in increased gene transcription.

*EZH2* is a histone methyltransferase component of the Polycomb group (PcG) of proteins. *EZH2* act as a transcriptional repressor and is a member of the Polycomb Repressive Complex 2 (PRC2). *EZH2* contains a C-terminal SET domain which enables the protein to exert its methyltransferase activity (Figure 5A). *EZH2*, or its homologue *EZH1*, forms part of the canonical PRC2 along with embryonic ectoderm development (*EED*), suppressor of zeste 12 homologue (*SUZ12*) and *RBBP4* (Figure 5B). *EZH2* is unable to carry out this enzymatic function alone, the interaction with *EED* and *SUZ12* enable gain of function. *EZH2* functions to catalyse the addition of a methyl group to histone H3 at lysine 27 (H3K27) creating mono/dimethylation of H3K27 (H3K27me1/2) (Figure 5C) [61]. The methylation of H3K27 serves as an anchorage point for the recruitment of other PcG proteins contributing to gene repression impacting development and regulation of stem cell lineage development [61].

*EZH2* is implicated in 2.59% of MDS cases, 0.94% of AML cases and 0.80% of MPN cases and generally confer a poor prognosis [62,63]. *EZH2* is the most frequently associated PRC2 member implicated in myeloid malignancies [9]. Majority of *EZH2* heterozygous mutations occur at tyrosine 641 (Y641) located in the SET domain resulting in gain of function mutations [64]. It is also worth noting that studies suggested the dual role of *EZH2* as an oncogene and a tumour suppressor. Tumour suppressor function of *EZH2* was identified as an interaction partner with *MLL-AF9/AML1-ETO9a* AML [65]. Basheer et al. discovered that the deletion of *EZH2* resulted in accelerated disease progression and reduced survival when occurring together with *MLL-AF9/AML1-ETO9a* suggesting that the presence of *EZH2* can function to provide full oncogenic effects; adding another layer of complexity to the multitude of effects *EZH2* contributes to cancer [65]. Mutations at Y641 can be a multitude of amino acid conversions; Y641F (Tyrosine-Phenylalanine), Y641N (Tyrosine-Asparagine), Y641S (Tyrosine-Serine), Y641H (Tyrosine-Histidine) or Y641C (Tyrosine-Cysteine). The mutants formed show increased affinity for H3K27me2 or H3K27me3 compared to WT-*EZH2* which expresses preference for unmethylated H3K27 or H3K27me1 [66]. Shen et al. have shown that the knockout of *EZH2* in embryonic stem cells reduces the methylation levels of H3K27me1/H3K27me2, however methylation is not removed in its entirety suggesting that *EZH1* can contribute to the methylation status within the genome (Figure 5D) [67]. Patients presenting clinically with a myeloid malignancy may display lower blast percentage (21–30%) [68]. At diagnosis patients displaying mutant *EZH2* may be slightly older than wild-type (59 years compared to 56 years) yet no differences are found between WBC counts, karyotype and ELN risk category of patients compared to wild-type [63].

**Figure 6:**
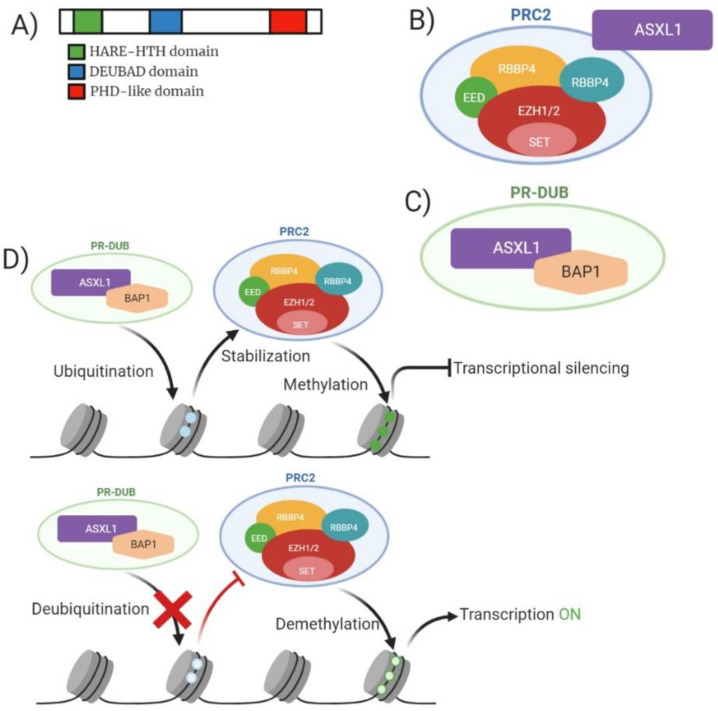
Analysis of *ASXL1* molecular structure, ASXL1/PRC2 structure, PR-DUB structure, and mechanism of action for wild-type and mutant *ASXL1*. (**A**) Domain structure of mammalian ASXL1 enzyme consisting of 1541 amino acid residues. Green highlighting HARE-HTH (HB1, ASXL1 restriction endonuclease helix-turn-helix). Yellow highlighting DEUBAD (deubiquitinase adaptor). Blue highlighting c terminal PHD domain. (**B**) Model of mammalian PRC2 complex with core subunits and association with *ASXL1*. (**C**) Model of PR-DUB complex with *BAP1* and *ASXL1*. (**D**) Mechanism of action for both wild-type and mutant *ASXL1* in relation to function with PRC2. Wild-type *ASXL1* contributes to ubiquitination of H2AK119 enabling stabilization of PRC2 leading to trimethylation of H3K27 resulting in transcriptional silencing. Mutant *ASXL1* causes deubiquitylation of H2SK119 preventing the stabilization of PRC2 resulting in reduced methylation of H3K27 resulting in increased gene transcription.

*ASXL1* is a human homologue of Drosophila additional sex combs gene and generally confers a poor prognosis, the gene is located on chromosome 20q11. [52]. Majority of the mutations that occur on the *ASXL1* gene are at exon 12 and are heterozygous [53]. Structurally *ASXL1* contains a conserved carboxyl-terminal PHD domain along with a HB1, *ASXL1* restriction endonuclease helix-turn-helix (HARE-HTH) and a deubiquitinase adaptor (DEUBAD) (Figure 6A). *ASXL1* is a member of a family of three identified genes (*ASXL1, ASXL2* and *ASXL3*) which regulate chromatin remodelling but remain poorly characterised. *ASXL1* functions within the PRC2 complex enabling cell differentiation via binding to the cohesion complex; controlling chromatin separation during cellular division. Scheuermann et al. highlighted the association between the polycomb-repressive deubiquitylase (PR-DUB) complex and its ability to deubiquitylate histone H2A at lysine 119 (H2AK119) (Figure 6B,C). This function can be accomplished by the components BRCA1-associated protein 1 (*BAP1*) and *ASXL1* [54]. The ubiquitination of H2AK119 enables stabilization of PRC1/PRC2 enabling efficient deposition of H3K27me3 (Figure 6D).

*ASXL1* is shown to be implicated in 11–29% of MPN and MDS cases, and 17% of AML patients. Genetic studies into the mutational profile of *ASXL1* suggest a higher occurrence of frameshift and nonsense mutations hence resulting in premature truncation of the protein upstream of the PHD finger. Truncation of this protein alters the functional state of the *ASXL1* protein and the association it holds with PRC2 preventing chromatin remodelling from being controlled- this also suggests a highlighted role of *ASXL1* being essential for roles of PRC1/2 (Figure 6D). In total, 50% of mutations in *ASXL1* pertain to the duplication of a guanine nucleotide (c.1924drpG) resulting in a frameshift (p.Gly646TrpfsX12) [69]. The loss or mutation of *ASXL1* alters the targets of the proteins including *HOXA* gene cluster. Expression of these gene clusters is directly increased by *ASXL1* loss resulting in poorer prognosis for AML due to increased inappropriate expression of genes implicated in differentiation [70,71]. Clinical presentation of patients with *ASXL1* are typically older male patients presenting with a more immature phenotype. There is a decrease in bone marrow and circulating blasts in these patients. Patients with mutated *ASXL1* experience lower complete remission (CR) than WT *ASXL1* patients (56% vs. 74%), with a 5-year EFS (15.9% vs. 29%). Patients with *ASXL1* are often associated with intermediate risk cytogenetics, and in this circumstance further association with trisomy 8 and del(7q)/-7 [72].

Similar to the previous mutations mentioned above mutated *ASXL1* co-occurs frequently with *IDH2^R140^* and *RUNX1* mutations while being rarely detected with *NPM1*, *FLT3-ITD* or *DNTM3A*. *ASXL1* is related in function to PRC2 but there is no evidence supporting mutual exclusivity with members of the PRC2 complex. It has also been proposed that ASXL1 can occur concomitantly with mutations in encoding signals (*JAK2*, *RAS*) and splicing proteins (*SF3B1*) [69]. ASXL1 interacts with binding partner *BAP1* however there is no evidence supporting mutations of *BAP1* being implicated in myeloid malignancies [69]. Patients possessing both *ASXL1* and *RUNX1* experience a poorer prognostic outcome. It has been suggested by Bera et al. that cooperation of *ASXL1* and *RUNX1* play a critical role in leukaemic transformation [73]. Patients with these cooperating mutations experience amplified proliferation of mutated cells with a progression block of differentiation and increased self-renewal. Mice transduced with these mutations heightened inhibition of DNA binding 1 protein inhibitor (*ID1*) expression. *ID1* is a transcriptional regulator that negatively regulates basic helix-loop-helix transcription factors inhibiting DNA binding and controlling transcriptional activity.

## 6. Targeted Epigenetic Therapies

Epigenetic alterations are an emerging field of cancer research and as such therapeutic options are limited. Hypomethylating agents are used as DNA methyltransferase inhibitors. Agents such as azacytidine and decitabine have shown success in the treatment of MDS [74]. Decitabine is a deoxycytidine analogue which can be incorporated into DNA during the S phase of replication. This results in decitabine binding to the DNA methyltransferase which obstructs the actions rendering it inactive. In single agent trials decitabine achieved complete remission (CR) rate of 24% with a median overall survival of 7.7 months for elderly patients unable to complete intensive standard care chemotherapy [75]. Azacytidine is a cytidine analogue primarily incorporated into RNA acting to inhibit RNA processing and functions. Azacytidine holds the ability to be converted to decitabine to exert its effects onto DNA also. As a single agent used to treat leukaemic malignancies azacytidine showed an OS of 12.1 months alongside a CR of 46.5% [76]. These agents have been shown to cause a reduction in global methylation marks in the genome creating a synthetically lethal phenotype leading to targeted cell death. *DNMT3A* has been shown to interact with and upregulate the *HOX* (homeobox) family of proteins. *HOX* proteins encode master regulators of embryonic development [77]. A range of agents which target *HOX* cluster genes; including *DOTL1* (EPZ-5676), *MLL*-menin interaction inhibitors and *MLL* inhibitors all of which showed knockdown of the cluster genes resulting in leukaemic cell death [78,79]. The use of these inhibitors shows how targeting biological processes impacted by the mutation can result in lessening of prognostic outcome on the patient. Growing knowledge of the interactions between epigenetic modifications suggest single agent therapies may not be adequate alone. Combination therapies have been the focus of many studies as the knowledge of epigenetics deepens. One such combination that showed promising results was azacytidine alongside Trichostatin A (TSA; HDAC inhibitor). Using these therapies together showed synergistic actions with the display of decreased cell viability and tumorigenic ability alongside an increase in anti-oncogenic expression of cells. In single agent experiments TSA showed no activity against methylated genes while azacytidine showed only weak induction of transcription [80]. A phase 2 clinical trial by Garcia-Manero et al. showed studies using azacytidine alongside a HDACi yielded a lower overall survival (OS) (19.1 months) and complete remission (CR) (52%) compared to monotherapy with azacytidine [81]. There are multiple active clinical trials currently combining these hypomethylating drugs with other compounds to assess combined impact. Trial NCT01869114 has combined Azacytidine with Sirolimus, an immunosuppressant commonly used to prevent kidney rejection, to assess the impact on MDS and AML patients unable to tolerate high dose chemotherapy (ClinicalTrials.gov identifier NCT01869114). Decitibine is currently being trialled alongside Ruxolitinib, a drug commonly used in the treatment of myelofibrosis, to assess the efficacy of an intensified conditioning regimen in patients considered high risk within haematological malignancies currently undergoing SC transplants (ClinicalTrials.gov identifier NCT04582604).

*TET2* can be used as a predictor for treatment response for MDS patients. Bejar et al. examined mutations for their association with overall survival. They found patients presenting with *TET2*, but lacking clonal *ASXL1* mutations, showed highest response rates (OS = 3.65). It was also shown that response to Azacytidine treatment post bone marrow transplant was increased in patients who lacked *TET2* [82]. It can be theorised that the lack of *ASXL1* removed the potential resistance to hypomethylating agents (HMAs) that can be conferred. While mutant *TET2s* mechanism of action upon sensitivity to azacytidine is still unclear. Some myeloid malignancies have been shown to display resistance to HMAs providing issues for patient treatment. *TET2* has been shown to act as a scaffolding protein to recruit components for DNA damage repair enabling another mechanistic avenue to exploit. *PARP* (poly ADP-ribose polymerase) inhibitors (PARPi) have provided an alternative avenue for potentially targeting these HMA-resistance neoplasms. It has been shown that *TET2* mutation results in downregulation of *BRCA2*- which can cause sensitisation to PARPi, *TET2* reduction has also been shown to impact upon homologous recombination (HR) [83]. This combination of cellular alterations means that the impairment of DNA damage along with treatment of DNA damaging chemotherapeutic agents may increase sensitivity to PARPi leading to better patient treatment for *TET2* mutant individuals [84]. A recent 2019 study conducted by Das et al. investigated the use of ascorbate (Vitamin C) treatment for *TET2* mutant AML patients [85]. Ascorbate is required, along with 2-oxygluterate, iron and oxygen for the function of *TET* dioxygenases enabling the active demethylation of DNA. It has been shown ascorbate deficiency mimics the impact of *TET2* loss by interacting with *FLT3* resulting in leukaemogenesis- ascorbate replenishment has been suggested to reverse these changes due to increasing *TET2* levels [85,86]. The action of high dose ascorbate treatment promoted DNA demethylation inducing stem cell maturation while suppression of leukaemic cells providing a potential alternative treatment options for AML patients [87]. Current clinical trials include NCT03682029 which investigates the role of oral vitamin C administration to potentially change the biology of low-risk myeloid malignancies (ClinicalTrials.gov identifier NCT03682029).

The use of hypomethylating agents against *IDH* mutations was assessed by DiNardo et al. [88]. When treated with decitabine and azacytidine as single therapies or in combination with current front-line therapy; there was no association found between *IDH* status and treatment response. The FDA approved Ivosidenib (AG-120) and Enasidenib (AG-221) target mutant *IDH1* and *IDH2*, respectively by blocking the production of 2-HG leading to reduced blast count and an increase in mature myeloid cells. A number of small molecule inhibitors are being developed to target *IDH* mutational products. This range of inhibitors are being directed against the hypermethylation of histones and DNA in cells containing mutant *IDH*. The over expression of 2-HG can also be targeted with AGI-5198 (selective inhibitor targeting *IDH1-R132*) by blocking the production of 2-HG by mutant cells resulting in the restoration of differentiation and proliferation in leukaemic cells [89]. Another inhibitor HMS-101 targeting *IDH1* has been developed and showed to induce cell death within bone marrow cells expressing mutant *IDH1* decreasing the mutant colony population [90]. The products of mutant *IDH* can be indirectly inhibited, where studies have suggested that glutaminase, an enzyme responsible for producing glutamine, can inhibit the growth of *IDH* mutant AML cells [91]. The inhibition of glutaminase alters the ability for cells to provide a production source for α-KG and 2-HG.

*EZH2* mutants showed concomitant occurrence with *RUNX1*, *ASXL1*, *NRAS* and *NPM1* suggesting that these mutations are associated with secondary AML. Deletion of the *EZH2* gene from cells resulted in aberrant cell cycle entry along with an increase in apoptotic cell death which promoted a potential therapeutic target for patients with this mutation [92]. Inhibition of *EZH2* is an emerging field in terms of myeloid therapy. Xu et al. investigated the role of UNC1999 (a dual inhibitor targeting both *EZH1* and *EZH2*) showing anti-leukaemic effects against *MLL-ENL* cells resulting in differentiation of general cell population and apoptosis of mutated cells [93]. More recently Scheepstra et al. highlighted the potential to optimise proteolysis-targeting chimera (PROTAC) mediated protein degradation has displayed anti-tumour effects against epigenetic regulators, e.g., *TRIM24* suggesting potential application to *EZH2* methyltransferase activity, which requires further work into understanding the mechanism of action for *EZH2* [94]. Swords et al. presented evidence supporting the interaction between *EZH2* and retinoic acid in AML patients [95]. They showed that *EZH2* mutations can impede the differentiation process by blocking retinoic acid (RA) differentiation due to the regulation of RA signalling by PRC2. This suggests the potential impact of epigenetic reprogramming (via inhibition by epigenetic inhibitors, i.e., EZH2i) sensitising cells to all-trans retinoic acid (*ATRA*) to produce a cumulative therapeutic result. There is evidence suggesting the combination of *EZH2* inhibitors (i.e., S-adenosylhomocysteine hydrolase inhibitor 3-deazaneplanocin A (DZNep)) and Panobinostat (histone deacetylase inhibitor) results in apoptosis of AML progenitor cells by inhibition of H3K27me3 along with deacetylation of histones [96]. Tazemetostat is a first in class oral *EZH2* inhibitor currently used in multiple clinical trials for patients with follicular lymphoma (ClinicalTrials.gov identifier NCT03456726 and NCT01897571) [97]. Information on all therapies mentioned are summarized within Table 1.

Therapeutic treatment targeting *ASXL1* mutations are limited, however patients with *ASXL1* and *RUNX1* mutations show poor response to HMAs hence causing reduced survival [98].

## 7. Future Perspectives, Prospects, Directions and Conclusion

The importance of the role of deregulated epigenetics in myeloid malignancies is rapidly evolving enabling improved patient stratification and treatment. A range of epigenetic modifiers regulating methylation of DNA (i.e., *DNMT3A, IDH1, IDH1, TET2*) or modification of histones (*EZH2* and *ASXL1*) provide a targetable mutation for a subset of patients which enhancing knowledge around concomitant expression of mutations in myeloid malignancies (Table 2). The highlighted effects of these mutations on methylation and histone alterations suggests further investigation into the response of the epigenome when treated with inhibitors for MPN, MDS and AML patients. Mutations in epigenetics can be associated with prognostic outcome with *DNMT3A* and *ASXL1* being associated with poorer outcomes highlighting the need for novel therapies targeting these high-risk aberrations. A number of these mutations remain vague in their functions and biological consequences for patients. The global impact upon the genome of these mutations needs to be evaluated further to understand how best to treat patients. Alterations in global chromatin architecture contribute to progression of myeloid malignancies and the interaction of concurrent mutations contribute to patient’s pathogenesis while fully elucidating precise interactions between proteins implicated. Recent advancements in the understanding of mutations in the (epi)genome have paved the way for epigenome exploration.

## Figures and Tables

**Table 1:** Table showing overview of mentioned drugs currently in development or approved for treatment of myeloid malignancies via epigenetic control.

Epigenetic Function	Compound	Gene Target	Approval Status
DNA methylation	Azacytidine	DNA (~20%) RNA (~60–80%)	Approved
	Decitibine	DNA	Approved
	Vitamin C	*TET2*	NCT03682029
	Ivosidenib	*IDH1*	Approved
	Enasidenib	*IDH2*	Approved
	AGI-5198	*IDH1*	Pre-clinical
Histone lysine methyltransferase	EPZ-5676	*DOTL1*	NCT03724084
Histone deacetylase	Trichostatin A (TSA)	*HDAC*	Pre-clinical
	Panobinostat	*HDAC*	Approved
Immunosuppressant	Sirolimus	mTOR signalling	NCT01869114
Kinase inhibitor	Ruxolitinib	*JAK2*	NCT04582604
Polycomb proteins	UNC1999	*EZH1*/*EZH2*	Pre-clinical
	Tazemetostat	*EZH2*	Approved

**Table 2:** Table summarizing the epigenetic modifiers including frequency within disease, biological activity, and concomitant mutations. R882- Arginine 882. 5-mC- 5-methylcytosine. 5-hmC- 5-hydroxymethylcytosine. 5-fC- 5-formylcytosine. F-caC- 5-carboxylcytosine (5-caC). α-KG- α-ketoglutarate. 2-HG- 2-hydroxyglutarate. PRC2- Polycomb repressive complex 2. H3K27- Histone 3 Lysine 27.

Gene	Frequency	Biological Activity	Concomitant Mutations	References
*DNMT3A*	~20% of de novo AML	De novo methylation of CpG dinucleotides creating 5mC. Majority of mutations occur as nonsense/frameshift mutations causing premature truncation of R882. R882 interacts with PRC1 causing downregulation of haematopoietic differentiation genes resulting an immature cell state which retains self-renewal capacity.	*NPM1, FLT3, IDH1/2*	[44,48,50]
*TET2*	~10% in AML, ~30% in MDS and ~50% of CMML	TET2 should convert 5-mC to 5-hmC/5-fC/5-caC eventually leading to demethylation. Deletion of *TET2* acts as a ’first-hit’ of mutational development in leukaemogenesis causing increased methylation and reduction of 5-hmC levels.	*IDH1/2, WT1, NPM1, FLT3-ITD, JAK2, ASXL1, CALR, SF3B1, RUNX1, DNMT3A*	[3,63,70,73]
*IDH1/2*	~20% of AML, ~5% of MDS	Global hypermethylation. Wild-type *IDH1/2* function to oxidase isocitrate to α-KG. Mutational hotspots for *IDH1* occur in Arg132 and *IDH2* hotspots occur within Arg140 or Arg172. Mutant *IDH1/2* cause production of 2-HG inhibiting *TET2* function. Interaction between *IDH* and *TET2* results in increased 5-mC resulting in impaired DNA damage repair mechanisms.	*TET2, NPM1*	[85,86]
*EZH2*	~3% of MDS, ~1% of AML and ~1% of MPN	Enzymatically active member of PRC2 and catalyses mono/dimethylation of H3K27. Mutations occurring at Y641 result in altered function, mutants show increased affinity for H3K27me2/ H3K27me3 enabling uncontrolled development of stem cell lineages due to loss of histone methyltransferase activity.	*RUNX1, ASXL1, NRAS, NPM1*	[93,94,95,99]
*ASXL1*	~20% AML and ~10–30% MPN/ MDS	Poorly defined PRC2 subunit which regulates chromatin remodelling and cell differentiation. Frameshift/nonsense mutations cause premature truncation targeting *HOXA* gene cluster resulting in altered differentiation.	*IDH2^R140^, RUNX1, JAK2, RAS, SF3B1*	[66,70,100,101]
